# Bilateral Multiple Focal Choroidal Excavations in a Patient with Paroxysmal Nocturnal Hemoglobinuria (PNH)

**DOI:** 10.1155/2019/7925737

**Published:** 2019-12-12

**Authors:** Ping Huang, Tal Ben Ami, Weiye Li

**Affiliations:** Department of Ophthalmology, Drexel University College of Medicine, 219 N. Broad Street, Philadelphia, PA 19107, USA

## Abstract

Focal choroidal excavation (FCE) is an unusual configuration characterized by focal thinning and pitting of the choroid in the absence of staphyloma or scleral ectasia. The etiology and clinical implications of FCE are currently unknown. We report a case of bilateral multiple FCEs in a patient with a long history of paroxysmal nocturnal hemoglobinuria (PNH). Focal choriocapillaris thinning and hyperreflectivity of the adjacent outer nuclear layer were revealed by spectral domain optical coherence tomography, which suggests the occlusion of the choriocapillaries and secondary degeneration of the above photoreceptors. We hypothesize that thrombophilia in the condition of PNH played an important role in the formation of FCE. Although there is no histopathological evidence to support the association between the choroid changes and PNH, this case may offer new clues as for the etiology of FCE.

## 1. Introduction

Focal choroidal excavation is an unusual configuration of the choroid detected by spectral domain optical coherence tomography (OCT), which shows focal thinning and pitting of the choroid with an intact sclera. This clinical entity was first described in a case report by Jampol and colleagues in 2006 [1]. Later, Margolis et al. [2] reviewed 12 patients with similar choroidal contour and recommended to use the term focal choroidal excavation (FCE) for these OCT image findings. They also hypothesized that the FCE is a congenital posterior segment malformation. After this unique choroid contour was recognized, FCE has been reported to be associated with several different pathologies, such as polypoidal choroidal vasculopathy [3–5], central serous chorioretinopathy (CSCR) [6], choroidal neovascularization [7–9], blunt trauma [10], multiple evanescent white dot syndrome [11], and best vitelliform dystrophy [12].

The published reports were based on images by different modalities including OCT, fluorescein angiography (FA), fundus autofluorescence (FAF), and indocyanine green angiography (ICG). However, there are no histopathologic studies to support them. The etiology and clinical significance of FCE are still unclear.

We are reporting an observation of FCE in a patient with paroxysmal nocturnal hemoglobinuria (PNH). PNH is a rare acquired stem cell disorder characterized by chronic intravascular hemolysis and hemoglobinuria, an increased risk of thrombosis, and a variable degree of bone marrow failure [13]. It has been previously reported that PNH may cause retinal vascular occlusions [14]. There is one report describing bilateral central serous retinopathy in a patient with PNH [15].

Herein, we present a case with bilateral multiple focal choroidal excavations in a patient with PNH.

## 2. Case Report

A 54-year-old Chinese man presented in the clinic for a routine two-year ophthalmic examination. Past medical history is PNH. The patient was diagnosed with PNH at the age of 22. He is on Eculizumab (anti-complement factor 5 antibody) infusion which he started in 2007.

**Figure 1:**
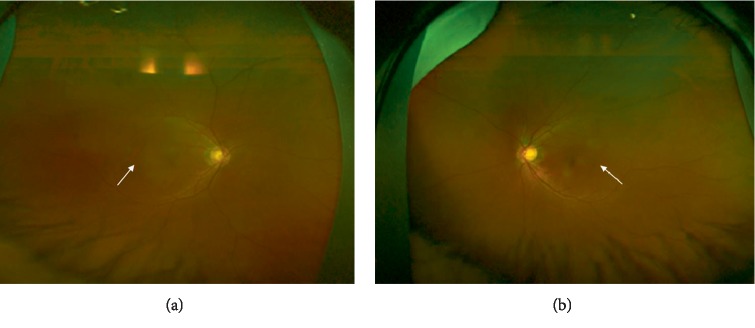
Fundus color photo of (a) right eye; (b) left eye. Subtle pigmentary changes temporal to the macula (arrow).

**Figure 2:**
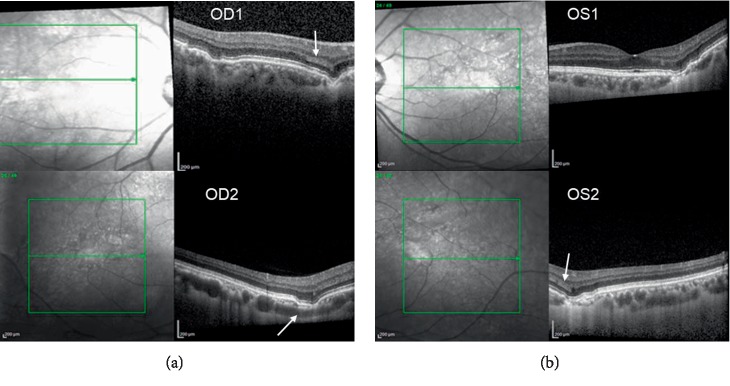
EDI-OCT scan of posterior pole of right eye (OD) and left eye (OS). OD1: Multiple focal choroidal excavations were showed in one horizontal scan. The outer nuclear layers (ONLs) conformed to retinal pigment epithelium (RPE) alterations within the excavation. And the RPE layer followed the contour of the choroidal excavation. There is no RPE detachment and no separation between the photoreceptor and the RPE. One area (arrow) of outer nuclear layers (ONLs) beside the excavation became hyperreflective with a density similar to that of the outer plexiform layer (OPL). OD2: Choriocapillaris in the site of excavation (Arrow) is thinner with adjacent large blood vessels changes comparing to the surrounding choroid. OS1: Multiple FCE without involving the fovea. OS2: Similar hyperreflective ONLs revealed in the left eye beside of the FCE lesion (arrow).

He was asymptomatic with best-corrected visual acuity of 20/40 on the right eye (spherical equivalent is +0.5), and 20/20 on the left eye (spherical equivalent is +0.25). Slit-lamp examination and intraocular pressures were normal. Posterior segment examination revealed subtle pigmentary changes temporal to the macula in both eyes (Figure 1). Enhanced depth imaging-OCT (EDI-OCT) of the macula was obtained and the entire posterior pole was also scanned. The OCT-detected multiple FCEs corresponded with the retinal pigmentary changes by infrared fundus photo in OCT (Figure 2). There was no FCE involving the foveal region. EDI-OCT showed the retinal pigment epithelium (RPE) layer complied with the contour of the choroidal excavation. And the outer nuclear layers (ONLs) conformed to the contour of the RPE within the excavation (Figure 2). There was no RPE detachment and no separation between the photoreceptor layers and the RPE. One area of ONL in proximity to the excavation demonstrated hyperreflectivity, similar to the reflectivity of the adjacent outer plexiform layer (OPL) (Figure 2 OD1 and OS2). The inner retinal layers from the OPL to the retinal nerve fiber layer were essentially undisturbed. Choriocapillaris thinning in the site of excavation was adjacent to large blood vessels as compared with the normal surrounding choroid layers without FCE (Figure 2 OD2). The sclerochoroidal junction was smooth and undisturbed without staphylomatous changes.

On FA, the FCE appeared to have normal fluorescence without leakage. No sign of neovascularization was found in the inner and subretinal space.

**Figure 3:**
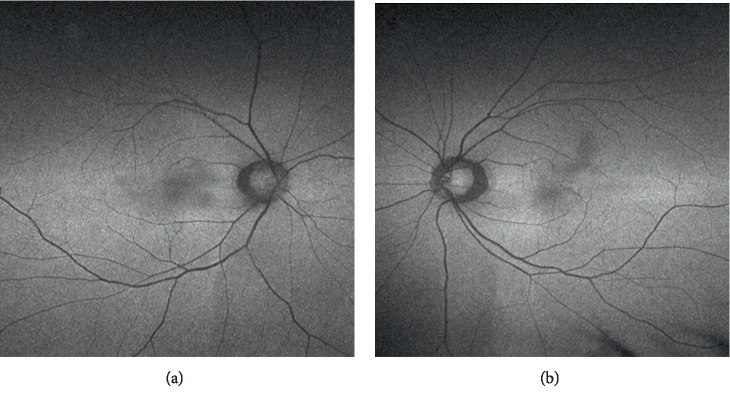
Autofluorescence photo of (a) right eye and (b) left eye showed irregular hyper and hypofluorescence in the macular area, which suggested some retinal pigment epithelium loss or damage.

FAF photos of both eyes showed irregular hyper and hypoautofluorescence in the macular area, which suggested some RPE loss or disturbance, which is corresponding to the locations of FCEs (Figure 3).

Humphrey Visual field testing in both eyes was performed twice. First was 24-2 Sita fast and the second was 10-2 Sita fast. But both of the visual field results were not reliable because of a high fixation loss.

The patient was observed without any intervention. The patient was followed up in 6 months with no changes of the symptoms, visual acuity, and the fundus exam.

## 3. Discussion

The case of bilateral FCEs we presented here is a 54-year-old Chinese male without any ocular disease. However, the patient has a significant systemic disease PNH. The formation of FCE in our patient might be associated with PNH.

Thus far, our knowledge and hypothesis as for the etiology of FCE is based solely on multimodal imaging findings, especially OCT, however no histopathological analysis of this phenomenon has been performed.

Increasing cases of FCE have been discovered and reported since this characteristic feature was obtained by OCT. Some studies have hypothesized that FCE is a congenital choroidal abnormality, which may also be associated with other types of focal structural defects within the choroid [1, 16, 17]. Two studies reported the acquired FCE was identified following treatment of choroidal neovascular membrane with antiendothelial growth factor (antiVEGF) therapy [16, 18]. Some authors believed FCE lesions could represent scarring of the choroidal connective tissue from a previous inflammatory process [6, 19]. But our patient does not have any past ocular history of inflammation or neovascular membrane.

Based on the morphology on the OCT image, FCE was classified into two types: one is the conforming type which shows the outer retina conforms to the choroidal excavation with preservation of the junction between the photoreceptor tips and the retinal pigmented epithelium. Second is the nonconforming type which corresponds to FCEs with a separation between the photoreceptor tips and RPE, forming a subretinal space [20]. Some studies showed that the nonconforming type was significantly associated with visual symptoms and central serous chorioretinopathy [16, 21]. And CSCR progressed to nonconforming FCE after the subretinal fluid resolved in three cases [22]. Our patient has multiple lesions of FCE in each eye, and all the lesions are of the conforming type, which is consistent with his negative FA findings and minimal disturbed visual acuity.

Our patient presents unique features that have not been previously described. Most of the previously reported cases featured a single, unilateral FCE lesion [23]. Our patient's FCEs are multiple and bilateral, diffusely involving the posterior pole. Since our current OCT device cannot reliably obtain scans from peripheral retina, it is unknown if our patient has any peripheral FCE. To our knowledge, in most of the previously reported cases of FCE, patients are young and healthy without obvious systemic medical problems which might link to FCE formation. This is the first case reporting the bilateral FCE in a patient with PNH.

So far, there is no report describing choroidal changes including FCE with PNH. However, some authors suggested that the FCE lesion may be related to choroidal vascular ischemic process according to the characteristic focal thinning of the choroid [23]. Thrombophilia remains the leading cause of mortality in PNH. Thrombosis most often involves unusual sites of the venous system (cerebral, mesenteric, and hepatic veins) [24]. One case of retinal vascular occlusion was reported as the primary manifestation of PNH [14]. In that case, FA demonstrated multiple occlusions of retinal veins in the inferior macular region of both eyes. Since no OCT was done in that case, it is unknown if the patient had FCE or not. Our patient had one prior hospitalization for blurry vision and right-sided paresthesias in 2014. Neurologic examination, CT head, and MRI brain with contrast at that time were all negative. Carotid Doppler test revealed mild bilateral plaques. This episode suggests that this patient could experience a thrombosis attack. However, no evaluation was done by ophthalmology at that time. It is conceivable that the focal choroidal thinning of FCE in our patient is the sequela of choriocapillaris occlusions. In addition, the alterations of choriocapillaris in the site of excavation may lead to degeneration of adjacent photoreceptors, which appeared as a hyperreflective band in ONLs adjacent to the excavation area (Figure 2 OD1 and OS2).

In this case report, we presented an interesting rare case of bilateral multiple FCE in a patient with a long history of PNH. Although there is no histopathological evidence to confirm the association between the choroid changes and PNH, this case may offer new clues as for the etiology of FCE. We encourage obtaining multimodal imaging, especially OCT, in patients with PNH who develop visual disturbances, to look for further evidence and association between these peculiar lesions and PNH. This approach may provide us with better understanding of the clinical course, evolution, and the relationship between the FCE and PNH.
